# Subjective social status is an important determinant of perceived stress among adolescents: a cross-sectional study

**DOI:** 10.1186/s12889-020-08509-8

**Published:** 2020-04-16

**Authors:** Pernille Bach Steen, Per Høgh Poulsen, Johan Hviid Andersen, Karin Biering

**Affiliations:** Department of Occupational Medicine, Danish Ramazzini Centre, Regional Hospital West Jutland – University Research Clinic, Gl. Landevej 61, DK-7400 Herning, Denmark

**Keywords:** Adolescents, Gender differences, Subjective social status, Perceived stress

## Abstract

**Background:**

Stress is an increasing public health problem, and the association between stress and subjective social status (SSS) among adolescents has received little attention. SSS in society have shown to be associated with perceived stress, but the association between SSS in school and stress has never been examined. The aim of this study was to explore the association between SSS and perceived stress in Danish adolescent boys and girls.

**Methods:**

Data was collected in 2017 in frame of The Danish Occupation of Children and Adolescents Cohort (FOCA cohort), where Danish 9th graders (age 15/16) from 1746 schools participated in a survey (4527 girls, 3654 boys, aged 15 to 16 years). SSS in society and SSS in school were the exposure variables, and the level of perceived stress was the outcome variable. Associations between SSS in school and in society separately with perceived stress was analysed using linear regression models stratified by gender and adjusted to social and health-related factors (e.g. neighbourhood safety, home characteristics, grade meaning, homework load, self-rated health, smoking, alcohol consumption).

**Results:**

The mean overall PSS score was 14.7; for girls the score was 16.3, and for boys it was 12.6. The analyses revealed a strong linear association between SSS, in both society and school, and perceived stress. The lower the SSS, the higher perceived stress. The associations were the same for both genders, but girls reported a higher level of stress than did boys.

**Conclusion:**

We found that girls reported a higher level of perceived stress than boys. Furthermore, we found a strong association between low SSS in society and especially SSS in school and a high level of perceived stress among Danish adolescents.

## Background

Stress is reported to be an increasing public health problem. The World Health Organization (WHO) estimates that by the year 2020, stress-related mental health conditions including anxiety and depression will be second on the list of the most burdensome diseases worldwide [1].

Stress is a response to a strain that a person may have difficulty coping with, and stress can be defined as a condition characterized by unevenness and tension [2].

The relationship between stress and later health problems among adults has been widely studied [3, 4], showing increased risks of cardiovascular diseases and depression and a poorer quality of life and well-being [2, 5, 6]. Despite a large amount of scientific work with in this area, research on stress among adolescents has not received much attention.

Studies among adults have documented the association between socioeconomic status (SES), measured by household income, parental educational level, or occupation, and health [7, 8]. A social gradient is present already in childhood, where children growing up in lower SES families tend to have poorer health than children of parents with higher SES [9, 10]. However, a social gradient appears to be less consistent in the adolescence [11]. This may be explained by the fact that this period of young people’s lives is characterized by a number of significant biological, cognitive, and social changes. This is also a period in which parental influence is decreasing, while personal autonomy is increasing [12–14]. These changes can lead to changes in personal behavior that can potentially have an impact on health [14, 15]. Due to this lack of consistency using objective measures of SES in relation to health among adolescents, the use of subjective social status (SSS) has been suggested as an alternative measure [13, 16], defined as a person’s own perception of social status. Studies have shown SSS to be associated with health outcomes, independent of SES in both adult and adolescent populations including mental health, psychopathological symptoms and psychological distress, and additionally SSS has been found to be a more sensitive predictor of adolescent health than objective SES [16–19]. Furthermore, self-perception of low social standing may be a psychological stressor that negatively alters health-related behaviors [20]. SSS among adolescents can be assessed as social status in society and social status in school by the youth version of the MacArthur Scale of Subjective Status [10]. The youth version of the MacArthur Scale is used to measure SSS in school to assesses adolescents perceived social status within their school by place themselves on the ladder according to where they believe they stand in relation to their classmates [10, 15]. Moreover, SSS in school is strongly associated with depressive symptoms [10]. Recently, findings in a study by Rahal et al. suggest that SSS in society is linked with differences in stress responsivity in late adolescence [21].

To date, previous research has primarily focused on the association between objective measures of SES and perceived stress among adolescents [13, 22]. One small study examined and found an association between SSS in society and perceived stress in an adolescent population [12], but to our knowledge no studies have examined a possible association between SSS in school and perceived stress among adolescents. A possible association is important for teachers as well as parents to be aware of in order to accommodate, respond, and react appropriately.

The aims for this study were to examine the current level of perceived stress among a representative sample of 9th grade students in Denmark, and to study the association between SSS, both in society and in school, and the level of perceived stress among girls and boys.

We hypothesises that low SSS both in society and in school is associated with higher level of stress than high SSS. Furthermore, we hypothesised that these associations were different between genders.

## Methods

### Design and population

The present study was a cross-sectional study based on data collected in the first survey in the establishment of a Danish national youth cohort: “The Danish Occupation of children and Adolescents cohort (the FOCA cohort)”. This cohort consists of a large sample of adolescents aged 15 to 16 attending 9th grade in schools across Denmark. The FOCA questionnaire contains questions capturing sociological, psychological, and health-related elements of importance for future education, work life, and well-being.

The FOCA cohort invited all eligible adolescents attending 9th grade at 1746 Danish schools, independent of school type [23]. Participants were found non-eligible if they were unable to answer the questionnaire due to cognitive challenges or severe reading and writing difficulties. All schools were asked to allocate one teaching session, but not every school would accommodate this request. The participation was 13,101 9th grade students from 650 schools in 97 of 98 municipalities in Denmark. Further description of the way the sample was collected is described by Lindholdt et al. [23].

The questionnaire was answered electronically and accessed by login on www.svar.foca.dk with the Uni-Login used in all private and public schools in Denmark as a personal identifier and log in for educational services. The average time to fill out the questionnaire was 31 min.

All data used in this study are self-reported and collected through the FOCA questionnaire during the first quarter of 2017. The FOCA questionnaire is available at http://foca.dk.

**Fig. 1:**
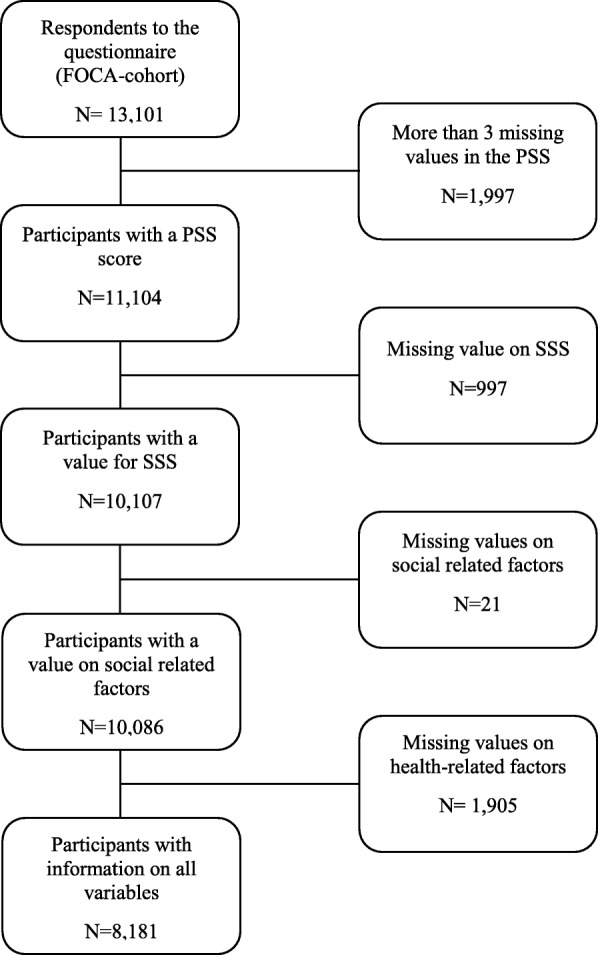
flow-chart – exclusion of participants

Of the 13,101 participants who took part in the survey, 1997 were excluded due to lack of information on the outcome variable (*n* = 11,104) and 997 were excluded due to missing information on the exposure variables SSS in society or in school (*n* = 10,107). Furthermore, 1926 participants were excluded due to missing information on the covariates. Based on this, the analyses were conducted with the participation of 8181 Danish adolescents (4527 girls and 3654 boys). See flow-chart (Fig. 1).

### Measures

#### Definition of Exposure

##### Subjective social status (SSS)

SSS was measured with the youth version of the MacArthur scale [10]. The MacArthur scale has proved to be a reliable indicator to link SSS to health outcomes in both countries with relatively large social inequalities [10, 16, 24] as well as the Nordic countries [25], characterized by small income inequalities due to a universal social policy based on a relatively high tax rate [26].

A Danish translated version of the youth version of the SSS scale was used in this study to measure the participant’s subjective perception of social status in society and social status in school. This instrument consists of two 10-rung ladders with different instructions for each of the ladders. We analysed the scale both as a continuous variable and as a categorized variable. The categorization of the study population was performed as three groups composed of the three lowest rungs (low SSS), the three highest rungs (high SSS), and the four in the middle (average SSS).

The instruction for the questionnaire used for the ladder measuring SSS in society was as follows:

“Imagine that this ladder pictures how the Danish society is set up. At the top of the ladder are the people who are the best off – they have the most money, the highest amount of schooling, and the jobs that bring the most respect. At the bottom are people who are the worst off – they have the least money, little or no education, no job or jobs that no one wants or respects. Now think about your family. Fill in the circle that best represents where your family would be on this ladder”.

The instruction for the questionnaire used for the ladder measuring SSS in school was as follows:

“Assume that the ladder is a way of picturing your school. At the top of the ladder are the people in your school with the most respect, the highest grades, and the highest standing. At the bottom are the people who no one respects, no one wants to hang around with, and have the worst grades. Where would you place yourself on this ladder? Fill in the circle that best represents, where you would be on this ladder.”

#### Definition of outcome

##### Perceived stress

The Danish version of the 10-item Perceived Stress Scale (PSS) [27] was originally developed by Cohen [28, 29] to measure current level of stress. PSS is a global stress measurement developed to assess whether one’s life is considered stressful. This is done by considering how unpredictable, uncontrollable and overloaded individuals find their lives [28]. PSS was used as an indicator for subjective perception of stress and asks for emotions and thoughts within the last month. The scale consists of 10 items, each rated on a five-point scale ranging from “never (0)” to “very often (4)” [28].

The total PSS score were calculated by reversing response to the positively stated items (items 4,5,7, and 8), and the sum across all items. For those missing one to three items, the mean of the other responses for that particular person was inserted as a single mean imputation. The PSS score ranged from 0 to 40, higher score indicated a higher level of perceived stress and has no defined or clinical cut-offs [28]. The scale was used as a continuous variable in the analyses.

In this study the PSS has shown a Cronbach’s alpha of 0.81, which implies internal reliability.

#### Potential confounders

Potential confounders were chosen á priori. Previous studies have found neighbourhood safety [30], self-rated health [10, 16, 17, 20, 25, 31], smoking [32], and alcohol consumption [32] to confound the association among adults, and furthermore, we included home characteristics, homework load, and grade meaning. Home characteristics were included as we consider two homes as proxy for living in a divorced family and thus a possible additional risk factor for stress [33]. Homework load and grade meaning were included as they in general are seen as risk factors for stress.

#### Social-related factors

Neighbourhood safety: Perception of neighbourhood safety was measured by an answer to the following statement: “I feel safe in my neighbourhood”. The four response categories (strongly agree, agree, disagree and strongly disagree) were merged and dichotomized into two categories (high or low neighbourhood safety), of which high neighbourhood safety involve the answers strongly agree and agree.

Home characteristics: number of homes was as in the survey dichotomy one or two homes, respectively.

Grade meaning: The adolescent’s perception of how important their own grades are was captured by the question: “How important are grades to you?”. There were four opportunities for response and we divided these into three categories (very important, important, or not/less important).

Homework load: The six categories of homework load (none, less than 1 h a week, 1–3 h a week, 4–6 h a week, 7–9 h a week and more than 10 h a week) were dichotomized (≤3 h/week or > 3 h/week).

#### Health-related factors

Self-rated health was in the questionnaire measured using a single item from the SF-36 on general health [34]. The five response categories were dichotomized into two groups (excellent/very good/good or fair/poor).

Smoking: Smoking habits were dichotomized (non-smoker/smoker). The participants who answered any of the three yes categories (less often than every week/not daily but at least once a week/daily) were categories as smokers.

Alcohol: The participants were asked about alcohol consumption within the last 30 days. Answers were dichotomized (≤2 days/month or ≥ 3 days/month).

### Statistical analyses

Descriptive analyses were conducted to identify the main characteristics of the participants, which are presented by complete cases and gender in Table 1. Data are presented as proportions and percentages or as means with standard deviations.

**Table 1:** Characteristics of the study population overall and stratified by gender

	Girls *N* = 4527	Boys *N* = 3654	Total *N* = 8181
Mean level of perceived stress (SD)	16.3 (6.6)	12.6 (5.9)	14.7 (6.5)
Subjective social status in society (%)			
Low	117 (2.6)	79 (2.2)	196 (2.4)
Medium	2767 (61.1)	2049 (56.1)	4816 (58.9)
High	1643 (36.3)	1526 (41.7)	3169 (38.7)
Subjective social status in school (%)			
Low	252 (5.6)	140 (3.8)	392 (4.8)
Medium	2519 (55.6)	1563 (42.8)	4082 (49.9)
High	1756 (38.8)	1951 (53.4)	3707 (45.3)
Neighbourhood safety (%)			
Low	114 (2.5)	60 (1.6)	174 (2.1)
High	4413 (97.5)	3594 (98.4)	8007 (97.9)
Home characteristics (%)			
1 home	3350 (74.0)	2676 (73.2)	6026 (73.7)
2 homes	1177 (26.0)	978 (26.8)	2155 (26.3)
Grade meaning (%)			
Very important	1686 (37.2)	811 (22.2)	2497 (30.5)
Important	2321 (51.3)	1915 (52.4)	4236 (51.8)
Not or less important	520 (11.5)	928 (25.4)	1448 (17.7)
Homework load (%)			
≤ 3 h a week	4199 (92.7)	3508 (96.0)	7707 (94.2)
> 3 h a week	328 (7.3)	146 (4.0)	474 (5.8)
Self-rated health (%)			
Fair/poor	281 (6.2)	165 (4.5)	446 (5.5)
Good/very good/excellent	4244 (93.8)	3486 (95.5)	7730 (94.5)
Smoking (%)			
Non-smoker	3720 (82.2)	3061 (83.8)	6781 (82.9)
Smoker	807 (17.8)	593 (16.2)	1400 (17.1)
Alcohol (%)			
≤ 2 days a month	4161 (91.9)	3207 (87.8)	7.368 (90.1)
≥ 3 days a month	366 (8.1)	447 (12.2)	813 (9.9)

The Spearman rank correlations test was applied to examine a possible correlation between SSS in school and SSS in society. The correlation between SSS in society and SSS in school in the ordinate scale was 0.34.

Statistical analyses were performed by linear regression models and stratified by gender. Initially, an unadjusted linear regression model was performed, estimating beta-coefficients to estimate the association between SSS in society and in school and level of perceived stress (Model I). The adjusted analyses were performed in three steps. Step one: Adjustments for the social-related factors were added to the analyses (Model II). Step two: Adjustments for the health-related factors were added model I (Model III). Step three: Adjustments for both social and health-related factors were carried out (Model IV). All potential confounders were included as categorical variables in the adjusted analyses. All analyses were stratified on gender and presented separately.

Analyses were carried out using complete cases to ensure comparability between crude and adjusted estimates. Additionally, an unadjusted linear regression model was performed, presenting beta-coefficients to estimate the associations between SSS in society and in school and level of perceived stress in the eligible population, to enable a comparison between the study population with the eligible population (Table 4). The models were checked by diagnostic plots of the residuals. Estimates were given with 95% confidence intervals (CI).

Data were analyzed using the statistical package Stata, statistical software version 15.10 (Stata Corporation, College Station, TX).

## Results

### Description of the study population

As shown in Table 1, Girls reported a significant higher level of stress than boys (*p* < 0.001). A larger proportion of boys than girls considered themselves to be in the category high SSS in society (*p* < 0.001). The same pattern was seen for SSS in school. Girls reported more often fair or poor general health than did boys (*p* < 0.001). Furthermore, girls also reported grades to be more important and to have a larger homework load than boys (*p* < 0.001). In contrast boys reported consuming alcohol more often than girls (*p* < 0.001).

When comparing the three exposure categories of SSS in society, the mean PSS score was 19.9 (SD ± 7.1) for low SSS in society, 15.2 (SD ± 6.4) for medium SSS in society, and 13.5 (SD ± 6.5) for high SSS in society. For the exposure categories of SSS in school, the mean PSS score was 20.6 (SD ± 7.1) for low SSS in school, 15.5 (SD ± 6.2) for medium SSS in school, and 13.2 (SD ± 6.4) for high SSS in school.

### The association between SSS and the level and perceived stress

Unadjusted and adjusted estimates of the linear regression models are presented in Table 2 (girls) and Table 3 (boys). The beta-coefficients in model I show the unadjusted association between SSS in society and in school and the level of perceived stress. In both genders, a lower SSS both in society and in school was associated with a higher level of perceived stress. These findings were consistent after adjustments in all regression models. The decrease in the beta-coefficients especially for low SSS both in society and in school in the adjusted analyses (Model II and Model III) indicates that the social- and health-related factors are confounding factors.

**Table 2:** The associations between SSS in society and in school and the levels of perceived stress among girls

	Level of perceived stress								
	Girls *N* = 4527								
	Model I		Model II		Model III		Model IV		
	β	95% CI	β	95% CI	β	95% CI	β	95% CI	Test for trend
Subjective social status in society									*p* < 0.01
Low	7.01	5.80–8.22	5.21	4.02–6.40	5.69	4.54–6.85	4.33	3.19–5.47	
Medium	1.62	1.23–2.02	1.01	0.61–1.40	1.40	1.03–1.76	0.90	0.53–1.28	
High (reference)	0		0		0		0		
Continuous scale	0.98	0.86–1.10	0.60	0.47–0.72	0.82	0.71–0.94	0.51	0.39–0.63	
Subjective social status in school									*p* < 0.01
Low	7.05	6.22–7.89	5.93	5.08–6.78	5.84	5.02–6.64	5.16	4.34–5.98	
Medium	1.76	1.37–2.15	1.60	1.21–1.99	1.74	1.37–2.11	1.66	1.28–2.04	
High (reference)	0		0		0		0		
Continuous scale	0.99	0.89–1.09	0.81	0.71–0.92	0.89	0.79–0.98	0.77	0.67–0.88	

Model I: Unadjusted

Model II: Adjusted for social-related factors: SSS in school or society, neighbourhood safety, home characteristics, grade meaning and homework load

Model III: Adjusted for health-related factors: Self-rated health, mental health, smoking and alcohol consumption

Model IV: Model II + Model III

**Table 3:** The associations between SSS in society and in school and the levels of perceived stress among boys

	Level of perceived stress								
	Boys *N* = 3654								
	Model I		Model II		Model III		Model IV		
	Β	95% CI	Β	95% CI	Β	95% CI	β	95% CI	Test for trend
Subjective social status in society									*p* < 0.01
Low	4.72	3.39–6.04	2.94	1.63–4.25	3.81	2.51–5.10	2.41	1.13–3.69	
Medium	1.23	0.84–1.62	0.74	0.35–1.12	1.13	0.76–1.51	0.70	0.32–1.08	
High (reference)	0		0		0		0		
Continuous scale	0.67	0.55–0.79	0.37	0.25–0.50	0.58	0.47–0.70	0.32	0.20–0.44	
Subjective social status in school									*p* < 0.01
Low	6.45	5.46–7.43	5.80	4.81–6.80	5.49	4.52–6.47	5.04	4.01–6.03	
Medium	1.94	1.56–2.32	1.79	1.40–2.18	1.97	1.59–2.34	1.85	1.47–2.24	
High (reference)	0		0		0		0		
Continuous scale	0.84	0.74–0.94	0.74	0.63–0.84	0.79	0.70–0.89	0.72	0.61–0.82	

Model I: Unadjusted

Model II: Adjusted for social-related factors: SSS in school or society, neighbourhood safety, home characteristics, grade meaning and homework load

Model III: Adjusted for health-related factors: Self-rated health, mental health, smoking and alcohol consumption

Model IV: Model II + Model III

Model IV shows the fully adjusted estimates. Girls with low SSS in society scored 4.33 points higher on the PSS scale than the reference group (High SSS in society). For boys this difference was slightly smaller, boys with low SSS in society scoring 2.41 points higher than the reference group. The differences in PSS scores were 0.90 points higher for girls and 0.70 points higher for boys when the medium SSS in society was compared with the reference group. Girls with a low SSS in school scored 5.16 points higher on the PSS scale than the reference group (High SSS in school). This difference was almost the same in the boys, i.e. 5.04 points. When medium SSS in school was compared with the reference group, the differences were 1.66 points higher for girls and 1.85 points higher for boys. The test for trend showed an exposure-response pattern; adolescents with low SSS had a significantly higher level of perceived stress. The *p*-value for interaction between gender and SSS in society was 0.02 for low status and 0.66 for average status, whereas for SSS in school is was 0.61 for low and 0.32 for average status, all compared to high status.

Analysis of the association between SSS on the ordinate scale and perceived stress revealed that for every step up the ladder measuring SSS in society the PSS score decreased with 0.51 points for girls and 0.32 points for boys. This decrease in PSS score was significantly larger when the exposure variable was SSS in school, showing that for every step up the ladder, the PSS score decreased with 0.77 points for girls and 0.72 points for boys.

## Discussion

The association between subjective social status and perceived stress in Danish adolescents was analysed using cross-sectional data of the FOCA cohort. The level of stress reported by the Danish adolescents was estimated to be 14.7 on the 10-item PSS and findings were unfavourable for girls in comparison to boys. Both lower SSS in society and lower SSS in school were associated with a significantly higher level of perceived stress in both genders. Adjusting on various social and health related variables slightly decreased this association.

The analyses also revealed that the association between SSS in society and the level of perceived stress was stronger for girls in comparation to boys. This did not apply to the association between SSS in school and the level of perceived stress, where the association was approximately the same for boys and girls. Furthermore, the analyses revealed that the association between SSS in school and the level of perceived stress was significantly stronger than the association between SSS in society and the level of perceived stress. These findings support previous studies showing that SSS, and especially SSS in school, is an important factor for adolescent health [16–19].

The higher level of perceived stress among girls compared to boys is consistent with previous findings [12, 13, 32, 35, 36]. This gender-specific difference might partly be explained because girls have greater psychological and emotional investments in interpersonal success than boys [36, 37]. Girls may also have a higher need for approval by peers and be more likely to blame themselves for relational problems [37]. It is also a possibility that PSS primarily captures stress symptoms in girls and not in boys, as recently stated by Trolle et al. [35].

The level of perceived stress among Danish adolescents estimated in this study is a little lower than estimated in the eNation Surveys from 2006 and 2009 from U.S [36]. There can be many reasons for why Danish adolescents reports lower levels of perceived stress than American adolescents, and a direct comparation is not possible, as Danish society and the norms and values of the Danes differ from Americans. Furthermore, the American adolescents in the above study were older than adolescents in this study and the adolescent population of this study was small.

The findings in this study, showing that lower SSS in society and in school are associated with a higher level of perceived stress, are similar to findings by Goodman et al. [12]. They found a strong association between lower SSS in society and a higher level of perceived stress among American adolescents, and suggest SSS as a more sensitive measures of social status than objective SES indicators, because they are the product of the integration of many selected factors of the individual in defining their relationship to the social environment [12]. One piece in the puzzle of how social inequalities in health arise is thus the link between social disadvantage and stress.

### Strength and limitation

The FOCA cohort was representative regarding gender, socioeconomic background and school type [23]. Responders represented all options in the Danish school system, which is a unique character and must be seen as a strength.

The sampling method in the FOCA was rather unconventional, and it was not possible to identify the actual size of the target population. Accordingly, a comparison between participants and non-participants is not possible.

More than one-sixth of the participants did not fill out questions on the PSS sufficiently to calculate a score. This fact could influence our findings regarding the level of perceived stress. If the most stressed adolescents tended not to answer the questions regarding perceived stress, it might have led to an underestimation. If their decision not to respond to PSS questions was associated with their assessment of SSS in society or in school, it could have led to selection bias. However, since the decision not to answer the questions was taken without the knowledge of the outcome in this study, it must be assumed that it could only led to non-differentiated selection. Perceived stress captured by PSS is an outcome within the last month. This may not necessarily indicate a constant stress load.

Exclusion of participants who had missing information on covariates may possibly have caused selection bias. The main reason for exclusion of participants were missing data on alcohol consumption and smoking habits. Therefore, additionally unadjusted analyses were carried out with all participants that had a PSS score and a value on SSS in society and SSS in school (Table 4). The exclusion did not change the estimates for the association between SSS in society or SSS in school and the level of perceived stress. Nevertheless, the excluded adolescents could have been different from the study population. This might be problematic and reduce the generalizability.

**Table 4:** Comparison of crude estimates of the study population and the eligible population

	Level of perceived stress							
	Girls				Boys			
	Study population = 4527		Eligible population = 5512		Study population = 3654		Eligible population = 4595	
	Β	95% CI	β	95% CI	β	95% CI	Β	95% CI
Subjective social status in society								
Low	7.01	5.80–8.22	6.43	5.37–8.22	4.72	3.39–6.04	4.70	3.58–5.83
Medium	1.62	1.23–2.02	1.57	1.21–1.93	1.23	0.84–1.62	1.24	0.89–1.59
High	0		0		0		0	
Continuous scale	0.98	0.86–1.10	0.94	0.83–1.05	0.67	0.55–0.79	0.66	0.56–0.77
Subjective social status in school								
Low	7.05	6.22–7.89	6.45	5.71–7.19	6.45	5.46–7.43	6.25	5.62–7.09
Medium	1.76	1.37–2.15	1.78	1.42–2.13	1.94	1.56–2.32	2.06	1.72–2.40
High (reference)	0		0		0		0	
Continuous scale	0.99	0.89–1.09	0.97	0.88–1.06	0.84	0.74–0.94	0.84	0.75–0.92

In this study, both exposure and outcome variables were based on self-reporting, posing a risk of common methods bias. In general, self-reported information may result in information bias and may lead to misclassification if participants systematically over- or underreport their SSS or level of perceived stress in the desire to appear in a certain way. As SSS and perceived stress are subjective appraisals which cannot be captured by objective measurements, both the MacArthur scale and PSS are suitable scales to use. SSS was asked in general and perceived stress was asked within the last month, therefore recall problems causing substantial bias seem unlikely.

Regarding the measuring of SSS in school, it should be noted that the instruction label may not be clear in a Danish context, since high social acceptance among peers and high grades may not always be correlated.

In the linear regression model, adjustments were made stepwise in major groups, which did not reveal which variable contributed the most to confounding. Sub-analyses showed that self-rated health and SSS (SSS in school if the exposure was SSS in society and vice versa) were the main contributors to confounding (data not shown).Additionally, the FOCA cohort did unfortunately not provide data to include previously found potential confounders in adolescents populations as objective measures of SES and parent SSS [12, 38].

Our hypothesis of different associations in boys and girls was only partially confirmed, and whether or not other variables could be modifiers was not tested. Other covariates could also be mediators, but this is difficult to determine in a cross-sectional study. This may be the case especially in the SSS in society, if this reflects the child’s upbringing that may had led to other problems, such as poor health and lifestyle habits.

The observed associations between SSS and perceived stress are not considered to be affected by severe bias by the limitations of this study. However, caution about causal interpretation is necessary due to the study designs, as both exposure and outcome variables were reported at the same time. Results in this study can with caution be transferred to adolescents in countries with school systems and social conditions similar to the Danish population.

## Conclusion

This study found girls to report a higher level of perceived stress than boys. Furthermore, this study found a strong association between low SSS in both society and school and a high level of perceived stress in both genders. This association was significantly stronger for SSS in school than for SSS in society. A significant gender difference was also found in the association between SSS in society and the level of stress, as the association was strongest among girls, but no gender differences was found in the association between SSS in school and the level of stress.

This study adds information to the small body of work on SSS and health outcomes. Additionally, findings suggest that both SSS in society and in school may be an important determinant of the level of perceived stress among adolescents.

## Data Availability

The data were used under license from the Danish Data Protection Agency, journal #1–16–02-461-16 and under their rules of data protection for the current study, and thus not publicly available.

## Abbreviations

FOCA: The Future Occupation of Children and Adolescents
PSS: Perceived Stress Scale
SES: Socioeconomic status
SSS: Subjective social status
WHO: The World Health Organization
